# Medication and trial duration influence postural and pointing parameters during a standing repetitive pointing task in individuals with Parkinson’s disease

**DOI:** 10.1371/journal.pone.0195322

**Published:** 2018-04-05

**Authors:** Deborah A. Jehu, Hiram Cantù, Allen Hill, Caroline Paquette, Julie N. Côté, Julie Nantel

**Affiliations:** 1 University of Ottawa, School of Human Kinetics, Ottawa, Ontario, Canada; 2 McGill University, Department of Kinesiology and Physical Education, Montréal, Québec, Canada; 3 Centre for Interdisciplinary Research in Rehabilitation (CRIR), Montréal, Québec, Canada; Purdue University, UNITED STATES

## Abstract

We aimed to determine the effects of levodopa medication on the performance of a repetitive pointing task while standing, and to investigate the optimal trial duration in individuals with Parkinson’s disease, and older adults. Seventeen individuals with Parkinson’s disease (5 freezers) and 9 older adults stood on force platforms for 30 s and 120 s while performing a bilateral repetitive pointing task, tracked by motion capture. Participants with Parkinson’s disease were assessed on and off medication and older adults were also assessed on separate days. The main findings were that: 1) on medication, participants with Parkinson’s exhibited greater center of pressure root mean square in the medial-lateral direction, greater velocity in the medial-lateral and anterior-posterior directions, and greater range in the medial-lateral direction than off medication; 2) longer trial durations resulted in greater center of pressure range in the medial-lateral and anterior-posterior directions and greater coefficient of variation in finger pointing on the least affected side; 3) Parkinson’s participants exhibited larger range in the medial-lateral direction compared to older adults; 4) off medication, freezers presented with less range and root mean square in the anterior-posterior direction than non-freezers; and 5) a correlation emerged between the freezing of gait questionnaire and pointing asymmetry and the coefficient of variation of pointing on the most affected side. Therefore, Parkinson’s medication may increase instability during a repetitive pointing task. Longer trials may provide a better depiction of sway by discriminating between those with and without neurological impairment. Individuals with Parkinson’s were less stable than older adults, supporting that they are at a greater risk for falls. The greater restrictive postural strategy in freezers compared to non-freezers is likely a factor that augments fall-risk. Lastly, the link between freezing of gait and upper-limb movement indicates that freezing may manifest first in the lower-limbs.

## Introduction

People with Parkinson’s Disease (pwPD) are approximately 3 times more likely to fall than their peers [1,2], with up to 50% of these falls resulting in injury [2]. Falling remains a major cause of increased healthcare services and costs in pwPD [3]. In fact, falls typically occur when medication is fully functioning [4], thereby underlining the importance of further investigating the role of medication on the control of posture in pwPD.

Postural instability is common with PD, whether on or off medication [5,6,7]. Some studies suggest that dopaminergic medication improves Berg Balance Scale scores [8] and results in less postural asymmetry [9]. However, other studies have highlighted no effect on balance [5], no effect on dual-tasking [10], as well as detrimental effects on balance [7,11,12]. Therefore, further research elucidating the influence of medication on posture is warranted.

Few studies have examined upper-limb movements in pwPD during sitting; though, the study of manual dual-tasks, such as pointing, has highlighted impairments in motor coordination [13–15]. Specifically, deficits in bimanual coordination have been observed when performing rapid and antiphase movements [13]. Other work has shown that pwPD undershoot a target and exhibit greater variability of arm motion during finger pointing [14,15], which may stem from an underestimation of the motor command and/or difficulty integrating proprioceptive feedback. Even fewer studies have explored the role of upper-limb movements on postural control during standing [16,17]. Poor spatial-temporal coupling of the center of mass and finger pointing [16] as well as ball-catching [17] has been delineated, perhaps because of deficits in anticipatory postural adjustments accompanied by attenuated arm speed. Imposing a task constraint to move the arm faster towards a target has been shown to confine the center of pressure (COP) to a smaller amplitude in pwPD, suggesting that the disease may alter the control of dual-tasking as well as the selection of appropriate postural strategies [17]. It is possible that exploring a repetitive pointing task (RPT) while standing may provide a better understanding of continuous motor coordination; however, this has not been previously explored.

Both freezing of gait (FOG) [7,18] and freezing of the upper-limbs [19,20] are apparent in a subgroup of pwPD. Freezing episodes have been defined as a temporary inability to generate effective movements [21]. Cognitive impairment [22] as well as an inability to generate symmetrical and rhythmical steps [23] appear to contribute to the mechanism(s) involved in freezing. Freezing seems to be more prevalent off medication [24], with fatigue [25], with prolonged disease duration [26], during anticipatory postural adjustments [27], and during dual-tasking [28]. Freezing during gait initiation, coupled with bradykinetic movements in shifting the center of mass to unload the stepping foot may also contribute to instability during gait [27]. Accordingly, it may be interesting to compare freezing of the upper- and lower-limbs to determine whether they originate from deficits in the same components of the motor control pathway.

Another area of understudied research is the investigation of optimal trial duration, as it is likely a factor in gaining an accurate depiction of posture. There appears to be no standard trial duration when examining posture on a force platform in pwPD, as previous research has employed trial durations such as 3–5 s [5,14], 30 s [9], and 45 s [29]. Nevertheless, empirical evidence suggests that the reliability of postural outcome measures increases with greater trial durations (i.e., 15 s, 30 s, 60 s, and 120 s) in young adults [30]. Similarly, other work has shown greater discrimination between single- and dual-task conditions as the length of the recording increased (i.e., 30 s, 60 s, and 300 s) in young adults [31]. Indeed, it is possible that a more accurate representation of postural control may be observed during longer trial durations in pwPD; however, no previous work has studied this question.

Therefore, the purpose of this study was to determine the influence of medication and trial duration on a bilateral RPT performed while standing in pwPD. A secondary objective was to determine whether pointing and postural parameters would be modulated in freezers compared to non-freezers, and whether FOG would be correlated with the kinematic characteristics of pointing. We hypothesized that pwPD would perform less pointing cycles due to bradykinesia, and would display greater pointing variability and pointing asymmetry (PA) while off relative to on medication as well as relative to older adult controls [15,32,33]. Additionally, it was thought that pwPD would exhibit greater COP variability, greater COP velocity, and larger COP range [6] while off relative to on medication, as well as compared to controls. Based on previous results in young adults revealing greater root mean square (RMS) and attenuated mean power frequency with longer trial durations [30], we hypothesized that a longer trial duration would lead to greater range of the COP and COP RMS in the ML and AP directions and greater CV in pointing. Finally, we hypothesized that further impairment in the upper- [19] and lower-limb parameters [7] would exist in freezers compared to non-freezers, and that the FOG questionnaire would be correlated with both PA and the coefficient of variation (CV) on the least and most affected sides as arhythmicity have been observed in pwPD [20].

## Method

### Participants

A group of 17 pwPD and 9 healthy age-matched adults participated in this study. All participants completed the testing protocol twice on separate days. Participants with PD were on dopaminergic medication during session 1, while they withdrew from medication for at least 12 hours for session 2. Both sessions were conducted at the same time of day to avoid within-day motor function fluctuations. Individuals with PD were recruited via flyers or word-of-mouth from the Health and Wellness program for pwPD at the Cummings Centre for Seniors in Montreal, Quebec, as well as through an announcement on the Quebec Parkinson Network website. pwPD were excluded from the study if: (1) they had any neurological, orthopedic, or muscular disorder, other than PD; (2) their score on the Montreal Cognitive Assessment (MoCA) test was lower than 26; (3) they had undergone deep brain stimulation surgery; (4) they were taking medication affecting balance (other than dopamine), (5) they had paresthesia, and/or (6) they had diabetes. The age-matched older adult controls were excluded from the study if they had any neurological, orthopedic, or muscular disorder, and/or criteria 2–6 as listed above. Participant characteristics are reported in Table 1. The same examiners clinically assessed each participant across testing sessions. All participants signed a consent form approved by the Research Ethics Board of the Centre for Interdisciplinary Research in Rehabilitation in Montreal.

**Table 1:** Participant characteristics (mean ± SD).

Outcome Measure	Older adults (*n* = 9)	Parkinson’s (*n* = 17)	Freezers (*n* = 5)	Non-freezers (*n* = 12)
**ge (years)**	68.2 ± 9.8	65.9 ± 6.8	67.0 ± 6.5	65.5 ± 7.2
**Sex (m/f)**	6/ 3	12/5	4/1	8/4
**Height (cm)**	168.7 ± 10.0	171.2 ± 7.8	171.1 ± 7.1	170.9 ± 8.3
**MoCA (units)**	28.0 ± 1.5	28.2 ± 1.5	28.4 ± 0.9	28.1 ± 1.7
**UPDRS (on)**	NA	23.1 ± 7.2	24.2 ± 4.4	22.6 ± 8.4
**UPDRS (off)**	NA	31.2 ± 9.2	31.3 ± 6.1	31.2 ± 10.2
**H & Y (on)**	NA	1.8 ± 0.6	2.0 ± 0.4	1.8 ± 0.7
**H & Y (off)**	NA	2.1 ± 0.7	2.3 ± 0.7	2.0 ± 0.7
**FOGQ (units)**	NA	3.4 ± 4.4	8.8 ± 4.4	1.1 ± 1.5
**Disease Duration (years)**	NA	6.1 ± 4.8	11.0 ± 6.0	4.1 ± 2.2
**Laterality (right/left)**	NA	8/9	3/2	5/7

Note: m/f: male/female; cm: centimeters; kg: kilograms; MoCA: Montreal Cognitive Assessment; UPDRS: Unified Parkinson’s Disease Rating Scale; on: on medication; off: off medication; H & Y: Hoehn & Yahr; FOGQ: Freezing of Gait Questionnaire.

### Clinical evaluation

During session 1, participants with PD completed 5 clinical evaluations: a medical history questionnaire, the Freezing of Gait Questionnaire (FOGQ), the Hoehn and Yahr (H & Y) scale, the Montreal Cognitive Assessment (MoCA) test, and the Unified Parkinson Disease Rating Scale Part 3 (UPDRS-III). The H & Y scale and UPDRS-part III were repeated in the off-state for the second visit. Control participants completed a medical history questionnaire and the MoCA test during session 1. Participants with PD were further categorized as freezers (*n* = 5) if freezing episodes were observed and/or participants answered positively to the FOGQ question #3 (score of 1 or greater): *“do you feel that your feet get glued to the floor while walking*, *making a turn or when trying to initiate walking”*.

### Experimental protocol

All participants performed a bilateral RPT during both sessions. A cylindrical touch-sensitive target (length: 6 cm, radius: 0.5 cm, response time: 130 ms; Quantum Research Group Ltd.) was placed in front of the participants’ midline at the sternum notch height located at a distance of 50% of the arm length. Participants pointed as fast as possible with both fingers kept pointed back and forth, one at a time, between the target and the sternum notch while standing with their feet positioned at approximately 25 cm apart on two adjacent force platforms. During the first session, target coordinates and feet placement were measured to ensure the same configuration was used in the second session. Reflective markers were placed on the touch-sensitive target, on the sternum as well as on the finger used to perform the task. A cycle was considered completed when the full distance between the target and the sternum was achieved. This was confirmed through the assessment of the kinematic data. Participants practiced the RPT twice for 20 s. This afforded sufficient practice while avoiding fatigue. Next, the experimental protocol consisted of performing one trial of the RPT at 30 s, and one trial at 120 s in a random order. At all times, participants were secured in a harness attached to the ceiling that did not hinder movement but was only used to catch them in the event of a fall.

### Data acquisition

Ground reaction forces and moments acting under the surface of the feet were measured by two triaxial strain gauge force platforms (AMTI OR6-7, AMTI, Inc., Watertown, USA). Kinetic data were sampled at a frequency of 1000 Hz and filtered at 6 Hz using a fourth-order low-pass Butterworth filter. The COP was detrended to remove any effect of participant position on the force plates. The dependent variables obtained from the force platform were: range, root mean square (RMS), and velocity of the COP in the medial-lateral (ML) and anterior-posterior (AP) directions.

A six-camera Vicon (4 MX3’s and 2 MX F20’s) motion capture system (VICON, Oxford Metrics Ltd., Oxford, UK) was used to record the positions and movements of both index fingers with a sampling frequency of 100 Hz. Pointing asymmetry (PA) was calculated as follows:

$$PA=\left|\ln\left(\frac{SSWT}{LSWT}\right)\right|\times 100$$

Where SSWT and LSWT corresponded to the shortest and longest mean pointing time between the arms, respectively [34]. The coefficient of variation (CV) of finger pointing on the least and most affected sides was calculated using the following formula:

$$CV=\frac{\sigma }{\mu }\times 100$$

Where *σ* and *μ* represented the standard deviation and mean pointing time, respectively.

### Data and statistical analyses

Three-way mixed analyses of variance were performed to determine if there were significant interactions and main effects of group (PD, Control), trial duration (30 s, 120 s) and medication (PD: Session 1 On, Session 2 Off; Control: Session 1, Session 2) with repeated measures on trial duration and medication for Range, RMS, velocity of the COP in the ML and AP directions, number of Pointing Cycles, PA, and the CV on the least and most affected sides. If Mauchly’s test of Sphericity was violated, the Greenhouse-Geisser correction was completed. To assess the association between freezing episodes in the lower- and upper-limb kinematics, Pearson correlation analyses between FOGQ and FOGQ question #3 with both PA and the CV on the least and most affected sides were performed. Finally, Mann-Whitney U tests were performed to compare postural and pointing parameters between freezers and non-freezers. Statistical significance was set to *α<*0.05 for the interaction and main effects. When applicable, post-hoc Bonferroni corrections were performed.

## Results

### Participant characteristics

Both participants with PD and controls performed the testing sessions 12 ± 11 days apart. Participants with PD and controls were matched for age, gender, height and cognition (Table 1). Freezers exhibited longer disease duration (*p*<0.05) and higher scores on the FOGQ (*p*<0.05) than non-freezers.

### Effect of duration and medication on postural control and kinematics

A three-way interaction effect between Duration, Medication and Group for RMS (*F*(1,22) = 7.04, *p*<0.05, *ƞ2* = 0.24) as well as Velocity (*F*(1,22) = 5.53, *p*<0.05, *ƞ2* = 0.20; Table 2) of COP in the ML direction emerged. Specifically, post hoc analyses revealed that RMS in participants with PD was larger on medication compared to off during the 120 s trials only (*p*<0.05). Velocity was larger on medication compared to off for both 120 s (*p*<0.05) and 30 s trials (*p*<0.05; Table 2).

**Table 2:** Center of pressure (COP) range, root mean square (RMS) and velocity in the medial-lateral (ML) and anterior-posterior (AP) directions during repetitive bilateral reaching task for 30 s and 120 s in controls and individuals with Parkinson’s disease (PD) on and off medication.

Kinetic Variable	Trial Duration (s)	Controls (n = 9)	PD on (n = 17)	PD off (n = 17)
**Range COP**_**ML**_ **(cm)**	120	5.5 ± 6.7	21.8 ± 13.3*^†^^§^	12.2 ± 4.4^*†*^ ^ϴ^
	30	4.4 ± 5.1	12.9 ± 7.0^#^^§^	9.7 ± 3.7
**Range COP**_**AP**_ **(cm)**	120	7.2 ± 4.2	12.6 ± 10.7*	10.0 ± 5.2
	30	6.6 ± 3.5	8.6 ± 8.1	7.9 ± 5.2
**RMS COP**_**ML**_ **(cm)**	120	2.1 ± 0.6	3.4 ± 1.7*	2.0 ± 0.8
	30	2.1 ± 0.7	2.3 ± 1.1	2.0 ± 1.0
**RMS COP**_**AP**_ **(cm)**	120	1.2 ± 0.8	2.0 ± 1.7	1.7 ± 0.9
	30	1.2 ± 0.5	1.6 ± 1.5	1.7 ± 1.0
**Velocity COP**_**ML**_ **(cm/s)**	120	5.9 ± 2.9	8.1 ± 5.6*	4.4 ± 2.2
	30	6.1 ± 3.3	7.5 ± 5.0*	4.9 ± 2.4
**Velocity COP**_**AP**_ **(cm/s)**	120	2.0 ± 0.8	4.1 ± 4.3*	2.0 ± 1.0
	30	2.4 ± 1.0	3.4 ± 3.5*	2.2 ± 1.2

* represents significant difference between PD on and PD off (*p*<0.05).

# represents a trend between PD on and PD off (*p* = 0.06).

§ represents a significant difference between PD on and Controls (*p*<0.05).

ϴ represents a significant difference between PD off and Controls (*p*<0.05).

† represents a significant difference between 30 s and 120 s (*p*<0.05).

Similarly, the two-way interaction effect between Duration and Group *(F*(1,22) = 6.12, *p*<0.05, *ƞ2* = 0.22; Table 2) for the Range of the COP in the ML direction revealed that when on medication, PD participants exhibited larger Range compared to controls for both 120 s (*p*<0.01) and 30 s (*p*<0.05) trials. Similar results were demonstrated off medication but only for the 120 s trials (*p*<0.05; Table 2).

A two-way interaction effect was shown between Medication and Group for COP Velocity in the AP direction (*F*(1,22) = 4.85, *p*<0.05, *ƞ2* = 0.18; Table 2). Further analyses showed larger velocity in the on compared to the off medication state for both 120 s (*p*<0.05) and 30 s (*p* = 0.05).

A two-way interaction effect between Duration and Group for COP Range in the ML direction was exhibited (*F*(1,22) = 6.12, *p*<0.05, *ƞ2* = 0.22; Table 2). When participants with PD were on medication, they revealed greater COP Range in the ML direction compared to off during the 120 s trials (*p*<0.05), while a trend was observed during the 30 s trials (*p* = 0.06). Range was also larger during the 120 s trials compared to the 30 s trials when on (*p*<0.01) and off medication (*p*<0.05).

In the same way, the two-way interaction effect of Duration and Group for the Range in the AP direction (*F*(1,22) = 17.52, *p*<0.01, *ƞ2* = 0.44) revealed larger Range in the 120 s trials compared to the 30 s both on (*p*<0.01) and off medication (*p*<0.05; Table 2) in participants with PD.

The main effect of Duration for pointing cycles *(F*(1,24) = 187.37, *p*<0.01, *ƞ2* = 0.89) revealed an average of 52 pointing cycles during the 120 s trials and 13 during the 30 s trials (*p*<0.01; Table 3).

**Table 3:** Number of reaching cycles, cycle asymmetry and coefficient of variation (CV) for the less and more affected side during a repetitive bilateral reaching task in controls and individuals with Parkinson’s disease (PD) on and off medication.

Kinematic Variable	Trial Duration (s)	Controls (n = 9)	PD on (n = 17)	PD off (n = 17)
**Reaching cycles (#)**	120	64.6 ± 23.6	51.1 ± 19.5*	53.4 ± 18.6*
	30	15.9 ± 7.4	12.8 ± 4.6	13.3 ± 4.8
**Asymmetry (%)**	120	1.5 ± 1.8	0.8 ± 1.7	1.3 ± 2.5
	30	1.9 ± 3.1	1.8 ± 2.6	0.9 ± 2.6
**CV less (cm)**	120	6.4 ± 4.8	9.8 ± 7.1	8.2 ± 5.7*
	30	5.4 ± 4.5	7.4 ± 6.4	5.4 ± 2.5
**CV more (cm)**	120	6.7 ± 3.7	8.0 ± 5.6	10.1 ± 6.4
	30	4.7 ± 4.8	7.7 ± 7.4	6.9 ± 7.7

* represents a significant difference between 30 s and 120 s (*p*<0.05).

A two-way interaction effect between Duration and Group for the CV on the least affected side (*F*(1,22) = 8.70, *p*<0.01, *ƞ2* = 0.30) was shown such that variability increased with duration during the off state (*p*<0.05; Table 3).

Statistical significance was not reached for the other metrics. No main effect of session or interaction effect between session and duration emerged for the control group.

### Effect of freezers compared to non-freezers on stability and reaching

The Mann-Whitney U test revealed attenuated Range and RMS of the COP in the AP direction in freezers compared to non-freezers in the off medication state during the 120 s trials (*p*<0.05), and a trend during the 30 s trials (*p* = 0.08; Table 4). No differences were shown on medication for kinetic and kinematic variables (Table 5).

**Table 4:** Center of pressure (CoP) range, root mean square (RMS) and velocity in the medial-lateral (ML) and anterior-posterior (AP) directions during repetitive bilateral reaching task for 30 s and 120 s in controls, as well as non-freezers and freezers on and off medication.

Kinetic Variable	Trial Duration (s)	Controls (n = 9)	Non-freezers on (n = 12)	Non-freezers off (n = 12)	Freezers on (n = 5)	Freezers off (n = 5)
**Range COP**_**ML**_ **(cm)**	120	5.5 ± 6.7	21.9 ± 15.9	15.5 ± 11.3	21.8 ± 4.9	11.6 ± 4.7
	30	4.4 ± 5.1	15.4 ± 15.5	13.0 ± 12.2	16.3 ± 5.6	10.1 ± 5.4
**Range COP**_**AP**_ **(cm)**	120	7.2 ± 4.2	12.8 ± 12.5	11.4 ± 5.9*	12.3 ± 6.1	6.6 ± 1.2
	30	6.6 ± 3.5	10.3 ± 11.0	9.7 ± 6.5	8.4 ± 2.9	5.9 ± 1.6
**RMS COP**_**ML**_ **(cm)**	120	2.1 ± 0.6	3.1 ± 1.9	2.3 ± 1.0	3.9 ± 0.9	2.0 ± 0.8
	30	2.1 ± 0.7	2.5 ± 2.0	2.5 ± 1.8	2.9 ± 0.9	2.0 ± 1.3
**RMS COP**_**AP**_ **(cm)**	120	1.2 ± 0.8	2.1 ± 2.0	1.9 ± 0.9*	1.9 ± 1.0	1.2 ± 0.2
	30	1.2 ± 0.5	2.0 ± 2.3	2.0 ± 1.2*	1.5 ± 0.6	1.3 ± 0.3
**Velocity COP**_**ML**_ **(cm/s)**	120	5.9 ± 2.9	7.0 ± 5.9	4.4 ± 2.6	10.6 ± 4.6	5.7 ± 3.0
	30	6.1 ± 3.3	8.7 ± 8.4	4.8 ± 2.5	9.0 ± 4.3	6.3 ± 3.4
**Velocity COP**_**AP**_ **(cm/s)**	120	2.0 ± 0.8	4.3 ± 5.1	2.1 ± 1.1	3.8 ± 2.1	1.9 ± 1.1
	30	2.4 ± 1.0	5.7 ± 7.0	2.3 ± 1.2	3.4 ± 1.7	2.2 ± 1.2

* represents a significant difference between freezers and non-freezers off medication (*p*<0.05).

**Table 5:** Number of reaching cycles, cycle asymmetry and coefficient of variation (CV) for the less and more affected side during a repetitive bilateral reaching task in controls, as well as non-freezers and freezers on and off medication.

Kinematic Variable	Trial Duration (s)	Controls (n = 9)	Non-freezers on (n = 12)	Non-freezers off (n = 12)	Freezers on (n = 5)	Freezers off (n = 5)
**Reaching cycles (#)**	120	64.6 ± 23.6	55.1 ± 21.9	55.3 ± 20.8	44.5 ± 7.1	47.0 ± 8.0
	30	15.9 ± 7.4	13.7 ± 5.1	14.2 ± 5.0	11.4 ± 2.6	11.5 ± 3.2
**Asymmetry (%)**	120	1.5 ± 1.8	0.8 ± 1.9	0.5 ± 0.7	0.7 ± 1.5	3.3 ± 4.0
	30	1.9 ± 3.1	2.0 ± 3.0	0.3 ± 0.2	1.2 ± 1.6	2.5 ± 4.8
**CV less (cm)**	120	6.4 ± 4.8	9.5 ± 8.1	7.9 ± 5.3	9.4 ± 4.5	10.4 ± 7.2
	30	5.4 ± 4.5	7.4 ± 7.2	4.8 ± 1.7	6.5 ± 4.1	7.1 ± 3.5
**CV more (cm)**	120	6.7 ± 3.7	7.8 ± 6.5	9.5 ± 5.2	7.7 ± 2.5	12.5 ± 8.8
	30	4.7 ± 4.8	7.6 ± 8.2	4.9 ± 2.4	7.3 ± 4.8	11.6 ± 13.0

Off medication, significant correlations between the upper-limb during the RPT and both the FOGQ and FOGQ question #3 were present. The FOGQ scores were related to PA in the RPT off medication for both the 120 s (*r* = 0.48, *p*<0.05) and 30 s trials (*r* = 0.69, *p*<0.01). The FOGQ scores were also correlated with the CV on the most affected side off medication during the 30 s trials (*r* = 0.75, *p*<0.01), but not during the 120 s trials (*r* = 0.08, *p*>0.05). Significant correlations were discovered between the FOGQ questions #3 and PA off medication during both the 120 s (*r* = 0.69, *p*<0.01) and 30 s trials (*r* = 0.51, *p*<0.05) as well as with the CV on the most affected side off medication during the 30 s trials (*r* = 0.51, *p*<0.05), but not during the 120 s (*r* = 0.31, *p*>0.05).

## Discussion

This was the first study to investigate the influence of medication and trial duration during a bilateral RPT performed in standing in pwPD. Notably, this study showed that: 1) on medication, participants with PD presented with greater instability relative to off medication, as exhibited by greater RMS of the COP in the ML direction, greater velocity of the COP in the ML and AP directions, as well as greater COP range in the ML direction; 2) the 120 s trials highlighted more postural stability deficit compared to the 30 s trials, as demonstrated by greater range of the COP in the ML direction and greater CV in pointing on the least affected side; 3) participants with PD presented more instability than the controls, as shown with a larger range of the COP in the ML direction; 4) freezers constrained their degrees of freedom relative to non-freezers off medication as observed by attenuated range and RMS of the COP in the AP direction; and 5) an association between upper- and lower-body freezing emerged off medication, as demonstrated by a correlation between the FOGQ and FOGQ question #3 to PA and CV on the most affected side.

### Medication and trial duration affect posture and pointing parameters in pwPD

Although medication has been developed with the intention to improve motor function, our study concurs with previous work suggesting that it may impair stability in some pwPD [7,12,35,36]. Larger ML sway amplitude and velocity have been associated with a reduced ability to effectively control posture and increased falls-risk [7,12,37,38,39]. Our results exhibited larger COP displacement in the ML direction during the longer conditions and greater COP velocity during both the 30 s and 120 s conditions on compared to off medication. This underlines the importance of assessing postural control in the ML direction. While there might be a trend, the lack of significant differences in pointing measures may suggest that the lower-limbs are differentially affected by medication than the upper-limbs. Perhaps the lower-limbs required greater cognitive involvement due to more advanced neurological impairment [40]. Alternatively, participants with PD could have prioritized the reaching task rather than securing postural stability [41]. Taken together, these results could be indicative of greater conscious control of lower-limb movements on medication, and highlights that pwPD may adopt an overall different postural strategy compared to controls. Future research should confirm the destabilizing effects of medication in large group of participants with different PD phenotypes, as alternative treatment prescription may be required, especially for those with poorer postural control.

Previous work has shown that repeated exposure to a testing protocol may result in inadvertent improvements in performance [42,43]. Marked improvements in stability were observed in the second session during the off-medication state relative to the first session on medication. Nevertheless, we are confident that these findings are a function of medication use, and not due to the repeated administration to the protocol, as there was no learning effects between sessions in the control group.

In line with our hypotheses, we found greater range in the AP direction and greater CV on the least affected side and greater RMS of the COP in the ML and AP directions during the 120 s compared to the 30 s trial durations. These findings corroborate previous work in young [30] and older adults [44], revealing that greater trial duration may afford a better representation of postural control and may better discriminate between older adult controls and those with neurological impairment. It is possible that participants with PD tightly controlled their posture during the first 30 s, but encountered greater instability as the trial progressed perhaps due to fatigue. However, fatigue alone cannot account for these findings. While an effect of trial duration emerged both the on and off medication for the COP range in the ML direction (Table 2), the COP range was almost twice as large on medication compared to off medication in the 120 s trials. This suggests that different postural strategies could have been adopted as a function of medication state. Future research should confirm and further explore the specific trial duration and medication contributions, as well as their interplay, to postural control in a larger sample of pwPD.

Interestingly, the CV was larger off medication during longer trials, but only on the least affected side. In contrast, previous research has displayed less variability on the least affected side during a seated supination and pronation task relative to the most affected side [45]. These differences may have emerged as the increased attention demand of standing compared to sitting [46] may have provoked our participants with PD to decrease the degrees of freedom of pointing, particularly on the most affected side, due to greater impairment. Despite the differing results, the current study, along with previous work [45], points to the importance of examining the least and most affected sides separately. We propose that examining the least affected side at longer trial durations may provide a better opportunity to discriminate between individuals with and without pathological conditions.

Dual-task paradigms have been largely used to examine the effects of automatic and controlled processing involved in postural stability in a variety of populations. Directing attention away from posture onto cognitive or motor tasks appears to result in more efficient postural control in young [47] and older adults [42,48]; however, it has shown a destabilizing effect in pwPD [49]. The current study exposed greater range and RMS of the COP in the ML direction in participants with PD compared to controls. In contrast, healthy young [47] and older adults [48] have demonstrated improved stability in dual-task contexts, likely due to the employment of unconscious, automatic, and efficient postural control. Our results suggest that pwPD likely adopt a different compensatory strategy, as automatic postural control may be impaired. The larger amplitude and variability of COP displacement are in line with previous studies reporting primarily lateral postural instability in pwPD [7,12,37,49]. Importantly, excessive postural sway, especially in the ML direction, has been correlated with a greater risk for falls in older adults and pwPD [50–54], and it therefore remains an important consideration for therapeutic interventions.

No statistical differences emerged across any of the kinematic measures of pointing between the PD and control groups. In contrast, one study examining standing and pointing revealed a correlation between increased arm movement displacement and greater COP amplitude in controls, with no differences in the PD group [16]. Notwithstanding, the task prioritization model postulates that improvements could occur in either or both tasks [55], as attention is limited, and one can only cope with a certain amount of information at any given time [56]. As such, the PD groups may have inappropriately allocated attention to the pointing task instead of prioritizing stability, which may explain the group differences in postural sway, but not pointing measures.

### Freezing influences both pointing and postural parameters

Interestingly, our results were contrary to our secondary hypothesis such that freezers demonstrated less range and RMS of the COP in the AP direction during 120 s trials relative to non-freezers off medication, and no statistical differences in pointing measures were observed between groups. Discriminating between freezers and non-freezers in the lower-limbs seems to be more common [7,18,34] than in the upper-limbs [19,20,34,57]. In contrast, other work has reported temporal but not spatial differences between freezers and non-freezers during a RPT [20]. Perhaps we did not observe freezing of the upper-limbs because of the type of upper-limb movement that we chose, given that the literature has shown that freezing is provoked by small movements of the upper-limbs as well as a lack of visual feedback [20].

Interestingly, although we did not observe freezing during the RPT, we found that the scores on the FOGQ and FOGQ question 3 were related to PA and the CV on the most affected side. Previous work suggests that there may be a link between lower- and upper-body freezing [20]. We propose that freezing in the lower-limbs may precede freezing in the upper-limbs, and may stem from similar underlying mechanisms. It is possible that the FOG and FOG question 3 may be used to predict future upper-limb freezing, which may first manifest on the most affected side due to greater impairment; however, this should be confirmed in a future longitudinal study with an adequate sample size.

Contrary to our hypothesis, the sway parameters of freezers off medication closely resembled those of healthy controls; however, these groups likely adopted different postural strategies. It is possible that dopamine depletion provoked freezers to assume a more constrained postural strategy to facilitate simultaneously performing the RPT. Future research should confirm whether pwPD undertake a stiffening strategy relative to young adults during a RPT with the use of electromyography. It is also important to reiterate that freezers presented with longer disease duration compared to non-freezers. Therefore, perhaps disease duration, as opposed to the presence of freezing, may have provoked differences between PD groups. Withal, given that freezing tends to manifest with longer disease progression, these two factors are difficult to independently separate.

## Conclusion

In summary, this study advocates that medication may play a role in postural instability; thus, it may be an important consideration for falls-risk assessments. The lack of differences between groups on the pointing measures could have been a function of an inappropriate task prioritization to pointing instead of postural stability. Longer trial durations seem to better characterize sway and discriminate between those with neurological impairment and controls. Participants with PD presented with greater instability relative to healthy controls, underpinning disease-related impairments in motor control. Lastly, greater postural instability as well as a link between lower- and upper-body freezing off medication emerged between freezers and non-freezers; therefore, separating these groups for statistical comparison may provide further insight into neurological deficits characteristic of PD.
